# Oxygen-carrying biomimetic nanoplatform for sonodynamic killing of bacteria and treatment of infection diseases

**DOI:** 10.1016/j.ultsonch.2022.105972

**Published:** 2022-03-02

**Authors:** Xiaorui Geng, Yuhao Chen, Zhiyi Chen, Xianyuan Wei, Yunlu Dai, Zhen Yuan

**Affiliations:** a Cancer Center, Faculty of Health Sciences, University of Macau, Taipa, Macau SAR, China; b The First Affiliated Hospital, Medical Imaging Centre, Hengyang Medical School, University of South China, Hengyang, Hunan, China; c Institute of Medical Imaging, Hengyang Medical School, University of South China, Hengyang, China; d Centre for Cognitive and Brain Sciences, University of Macau, Taipa Macau SAR, China

**Keywords:** Sonodynamic therapy, Metal organic frameworks (MOFs), Infection diseases, Nanomedicine, Hemoglobin, Multidrug-resistant (MDR) bacteria

## Abstract

Among various novel antimicrobial therapies, sonodynamic therapy (SDT) exhibits its advantages for the treatment of bacterial infections due to its high penetration depth and low side effects. In this study, a new nanosonosensitizer (HFH@ZIF-8) that loads sonosensitizer hematoporphyrin monomethyl ether (HMME) into zeolitic imidazolate framework-8 (ZIF-8), was constructed for killing multidrug-resistant (MDR) bacteria and treatment of *in vivo* infection diseases by SDT. In particular, the developed HFH@ZIF-8 exhibited enhanced water-solubility, good biocompatibility, and improved disease-targeting capability for delivering and releasing HMME and ablating the infected lesion. More importantly, the presence of oxygen-carrying hemoglobin for HFH@ZIF-8 can offer sufficient oxygen consumption by SDT, augmenting the efficacy of SDT by improving ROS generating efficiency against deep tissue multidrug-resistant bacterial infection. Therefore, this study paves a new avenue for treating infection disease, particularly for antibiotic resistant bacterial infection.

## 1 Introduction

Bacterial infection signifies a severe risk for public health [1]. In particular, increased infections due to the multidrug-resistant (MDR) bacterial species have significantly reduced the therapeutic efficiency of antibiotics [2].Therefore, it is essential to explore alternative treatment strategies including the development of bactericidal agents to eradicate bacterial infections and eliminate the occurrence of novel drug resistance. Among all the new therapeutic strategies developed to resolve drug resistance issues, photo-responsive photodynamic therapy (PDT) is an attractive antibacterial approach, which is able to kill bacterial pathogens by generating highly cytotoxic reactive oxygen species (ROS) through photochemical reactions [3], [4], [5], [6]. For phototherapy like PDT and PTT, the penetration depth for visible light or near-infrared light in the first window is around 1 cm. By contrast, sonodynamic therapy (SDT) exhibits its unbeatable advantages in killing bacteria, which uses ultrasound transducers and sonosensitizers to generate ROS [7], [8], [9], [10]. In particular, compared to that of PDT, the penetration depth for SDT can be up to 10 cm, indicating that non-invasive SDT can be carried out to treat deep infections.

To date, a bunch of sonosensitizers including porphyrin-based compounds, curcumin, and rose bengal have been developed for SDT [11], [12], [13], [14]. However, most of them are highly hydrophobic, which allows them to be easily eliminated from the blood circulation and results in the insufficient accumulation of sonosensitizers in the microenvironments of disease areas. Meanwhile, hypoxia is another inherent phenomenon that affects the treatment efficiency of SDT. During SDT, the continuous ROS production strongly depends on the sufficient oxygen supply to the infected areas [15], [16], [17]. However, most bacterial infections can cause a severely hypoxic microenvironment, which is able to significantly worsen the therapeutic effect. More importantly, the rapid consumption of oxygen during SDT will further deteriorate the hypoxia lesion areas and inhibit the killing effect of SDT on bacterial cells.

Interestingly, the latest advances in nanotechnologies and materials chemistry have offered new strategies for SDT in the targeted delivery of sonosensitizers and regulation of hypoxic microenvironment [18], [19], [20], [21], [22], [23], [24], [25], [26]. In particular, metal organic frameworks (MOFs), as a new type of porous material composed of metal ions connected with organic binding ligands, have received extensive attentions as a multifunctional agent for disease theranostics, particularly for antibacterial applications2 [7], [8], [9], [10], [11], [12], [13], [14], [15], [16], [17], [18], [19], [20], [21], [22], [23], [24], [25], [26], [27], [28], [29]. Importantly, zeolitic imidazolate framework-8 (ZIF-8) as a porous MOF has exhibited its potential in killing bacteria either by releasing of its zinc ions or by the delivery of antibacterial agents [30].

**Fig. 1:**
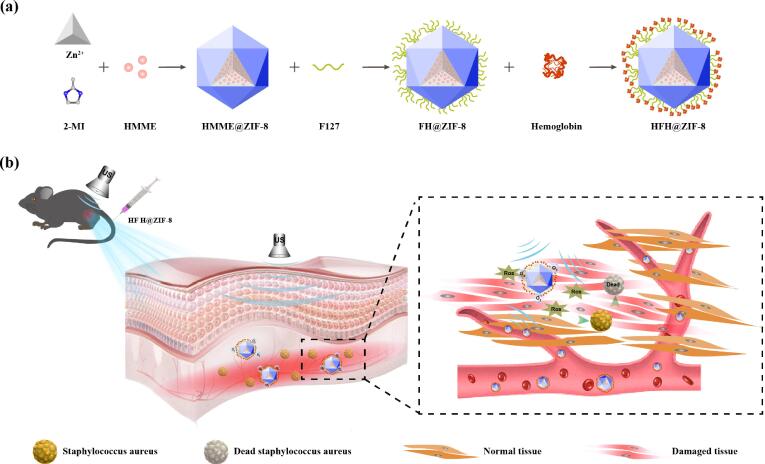
Schematic illustration of the hemoglobin-modified metal organic framework (HFH@ZIF-8) applied for sonodynamic therapy of deep bacterial infections.

In this work, a multifunctional nanotherapeutic platform (HFH@ZIF-8) was developed for bacterial killing and the treatment of infection diseases, which can augment the efficacy of SDT against deep tissue MDR bacterial infection through hemoglobin carrying oxygen. For the novel hydrophilic nanoplatform, the sonosensitizer hematoporphyrin monomethyl ether (HMME) was firstly loaded into the ZIF-8 nanoparticles. And then the HFH@ZIF-8 nanoplatform was constructed by modifying H@ZIF-8 nanoparticles with the F127 and hemoglobin through electrostatic incorporation (Fig. 1) [31]. Our pilot results demonstrated that HFH@ZIF-8 exhibited enhanced biocompatibility and high drug loading efficiency.

Published studies have demonstrated that proteins have advantages in the biomimetic synthesis of nanostructures, including green synthesis characteristics, functional integration and high biocompatibility [32], [33]. In this study, the reason we use hemoglobin is because of its natural biocompatibility to extend the circulation time of nanoparticles in the body and achieve more accumulation in inflammation sites [33], [34], [35]. In particular, the presence of oxygen-carrying hemoglobin can offer sufficient oxygen consumption by SDT, and effectively alleviate the hypoxic microenvironment of the bacterial infection areas [33], [36]. Meanwhile, *in vivo* fluorescence imaging illustrated the efficient accumulation of HFH@ZIF-8 in bacterial infective myositis sites in mice legs, whereas continuous monitoring of HFH@ZIF-8-mediated SDT by magnetic resonance imaging (MRI) revealed that the effective therapeutic progression of deep bacterial myositis induced by methicillin-resistant Staphylococcus aureus (MRSA). Basically, HFH@ZIF-8 exhibited an overall high efficacy in killing bacteria, indicating that they are a new promising nanoagents towards controlling bacterial infections and resolving the drug resistance.

## 2 Materials and methods

### 2.1 Preparation of HFH@ZIF-8

A balance was used to weigh out 162 mg of dimethylimidazole (Sigma), 73.32 mg of zinc nitrate hexahydrate (Sigma), and 1 mg of HMME (Di-Bai Chemical Technology Co., Ltd.). They were then totally dissolved in 15 mL of methanol, stirred at room temperature for 20 min, and then centrifuged for 5 min to obtain a pellet (H@ZIF-8). The precipitate was resuspended in ethanol. And then the F127 solution (5 mg/mL) and tH@ZIF-8 nanoparticles were mixed, stirred for 2 h, and centrifuged again to produce the precipitate (HF@ZIF-8). The precipitate was dissolve with water and mixed with the hemoglobin solution (1 mg/mL) and stirred overnight on ice. The free hemoglobin was further removed by centrifugation. The nanoparticles solution was oscillated on the shaker while introducing pure oxygen, which was repeated for 3 times until fine bubbles emerged from the liquid surface and centrifuge to generate HFH@ZIF-8.

### 2.2 Characterization of HFH@ZIF-8

The diameter and zeta potential of HFH@ZIF-8 nanoparticles were measured with DLS (NanoZS 90, Malvern, USA). The morphology of FH@ZIF-8 and HFH@ZIF-8 was respectively imaged by transmission electron microscope (TEM). The hemoglobin modification was accessed by using sodium dodecyl sulfate polyacrylamide gel electrophoresis (SDS-PAGE). The ultraviolet absorption spectrum and fluorescence spectrum of various categories of nanoparticles were measured and quantified by using ultraviolet spectrophotometer and fluorescence spectrophotometer, respectively. The 1,3-Diphenylisobenzofuran (DPBF) was used as the singlet oxygen probe to detect the ROS production of HFH@ZIF-8-based SDT. The concentration of DPBF used for the present work was 8 μM. After DPBF was mixed with the nanoparticle solution, the samples were irradiated by low intensity focused ultrasound (0.5 MHz, 1.2 W/cm2, 50% Duty cycle, 10 min). The ultraviolet absorption peak of DPBF was measured to evaluate the presence of singlet oxygen.

### 2.3 In vitro Anti-Bacterial experiment

To detect MRSA viability after HFH@ZIF-8-mediated SDT, MRSA bacteria (10^7^ CFU/mL) were inspected by different treatment strategies: (1) control group (PBS solution), (2) US group, (3) FH@ZIF-8 group (100 μg/mL), (4) HFH@ZIF-8 group (100 μg/mL), (5) FH@ZIF-8 + US group and (6) HFH@ZIF-8 + O_2_ + US group. After US irradiation (0.5 MHz, 1.2 W/cm^2^, 50% Duty cycle, 10 min). The ultrasound parameters used were based on the previous studies [37], [38]. The mixture solution was serially diluted and plated on lysogeny broth (LB) agar plates. The number of bacterial colonies were then after 37 ℃ culture for overnight. The home-made ultrasound irradiation setup consisted of a signal generator (JL-JLCJ-01-01, Yiwu Jielian Electronic Technology Co., Ltd.), a power amplifier (JL-JLCJ-01-02, Yiwu Jielian Electronic Technology Co., Ltd.), and a focused ultrasound transducer (1206260, Olympus) with the focus size of 0.5 cm^2^ and focal length of 10 cm.

### 2.4 Live/dead bacterial staining assay

The live/dead bacterial staining assay was used to inspect the bacterial killing capacity of HFH@ZIF-8-mediated SDT. MRSA solution (10^7^ CFU/mL) was randomly divided into 6 treatment groups: control group, US group, FH@ZIF-8 group (100 μg/mL), HFH@ZIF-8 group (100 μg/mL), FH@ZIF-8 + US group and HFH@ZIF-8 + O_2_ + US group. The treated bacterial solutions were collected at 5000 rpm for 5 min, and then resuspended in saline, and stained with a mixture of SYTO 9 and PI solution for 30 min. The stained bacteria solution was dropped on the glass slide for fluorescence imaging with a fluorescence microscope. To examine the potential biological mechanism associated with SDT-triggered bacteria death, scanning electron microscopy (SEM) was utilized to detect the bacterial morphology changes.

### 2.5 Animal studies

The used female C57 mice (4–6 weeks old) purchased from Vital River Laboratory Animal Technology Co (Beijing, China) were used to establish MRSA-induced myositis-bearing mice model. 50 μl bacterial cell suspension were then injected into the right leg of the mice and inflammation progression was monitored by MRI. SDT t was performed at 48 h post-injection. Before MRI, mice were anesthetized by the isoflurane gas. After the *in vivo* experiments, all mice were euthanized by using carbon dioxide asphyxiation. All animal experiment tests were approved by the Institutional Animal Care and Use Committee (IACUC) of University of Macau.

### 2.6 *In vivo* distribution evaluation

The FH@ZIF-8 and HFH@ZIF-8 solution was respectively injected into the myositis-bearing mice via the tail vein at the HMME dose of 5 mg/kg in 200 μl solution (n = 3). At the given time points, the mice were imaged by IVIS system (PekinEmer) to evaluate the biodistribution of nanoparticles. Myositis-bearing mice were sacrificed 24 h post-injection and the organs of the mice including heart, lung, liver, kidney, spleen and infected leg were extracted for ex vivo fluorescence imaging. In addition, the fluorescence intensity of organs was collected to demonstrate the nanoparticles accumulation.

### 2.7 *In vivo* SDT of bacteria infection

The myositis-bearing mice were divided into 6 treatment groups randomly (n = 5): (1) control, (2) US, (3) FH@ZIF-8, (4) HFH@ZIF-8, (5) FH@ZIF-8 + US, (6) and HFH@ZIF-8 + O_2_ + US groups. PBS was injected intravenously into myositis-bearing mice for both the control and US treatment groups. The mice in other groups were injected intravenously with nanoparticles at the HMME dose of 5 mg/kg in 200 μl solution. Regarding the US, FH@ZIF-8 + US and HFH@ZIF-8 + US treatment groups, US irradiation was conducted 24 h and 48 h post injection and the ultrasound parameter used were: frequency = 0.5 MHz, acoustic intensity = 1.2 W/cm^2^, duty cycle = 50%, and exposure time = 10 min. MRI (Aspectimaging-M3, Aspect) was used to monitor the myositis development every 4 days during the healing process. MRI parameters used for this study were: TR = 3250 ms, TE = 68.66 ms, ETL = 12 ms, Dwell time = 25 ms, and Pixel bandwidth = 166.667 Hz/pixel. The calculation formula of abscess area ratio was: ratio = (area of abscess/ healthy opposite leg area) × 100%. On the fifteenth day of the treatment, the infected muscle was collected, weighed and homogenized in sterile PBS in ice. The tissue grinding solution was diluted from 10,000 times with PBS. 100 μl tissue grinding solution was then plated on the LB plate. The colonies generated after 12 h incubation at 37℃ was counted. In addition, H&E staining was used to capture the histopathological changes of infected muscle on the fifteenth day. Meanwhile, the serum TNF-α level was detected on the fourth day and fifteenth days via ELISA, respectively.

### 2.8 Safety evaluation

To evaluate the biosafety of FH@ZIF-8, blood routine indexes, blood biochemical indexes and H&E staining of organs were tested respectively. The C57 mice were randomly divided into 6 groups (n = 3) including the control group, US group, FH@ZIF-8 group, HFH@ZIF-8 group, FH@ZIF-8 + US group and HFH@ZIF-8 + O_2_ + US treatment group. The treated mice were sacrificed 2 days after ultrasound irradiation. Blood samples (0.8 mL for each mouse) were collected by eyeball extirpating. The organs were further extracted to perform H&E staining.

### 2.9 Statistical analysis

Data are presented as mean ± S.D. Statistical analysis was carried out with one-way analyses of variance (ANOVA) for multiple comparisons. All statistical analyzes were performed with SPSS and all tests with P values of < 0.05 were considered as significant (***P < 0.001; **P < 0.01; *P < 0.05;).

## 3 Results and discussion

### 3.1 Preparation and characterization of HFH@ZIF-8

**Fig. 2:**
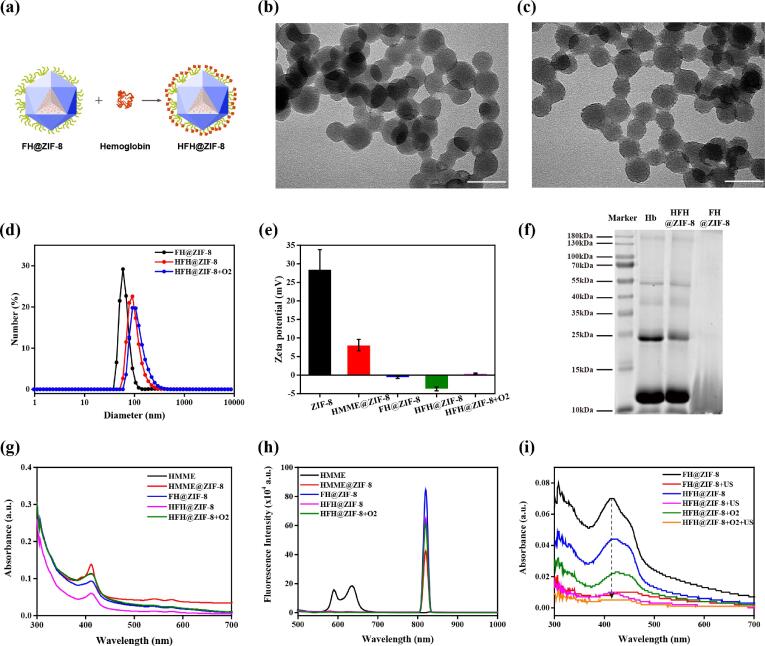
(a) The surface of FH@ZIF-8 was modified with hemoglobin to produce the biomimetic nanoplatform HFH@ZIF-8. TEM images of FH@ZIF-8 (b) andHFH@ZIF-8 (c). Scale bar: 50 nm (d) Hydrodynamic diameters of FH@ZIF-8, HFH@ZIF-8 and HFH@ZIF-8 + O_2_ in H_2_O measured by DLS, respectively. (e) Zeta potential of ZIF-8, HMME@ZIF-8, FH@ZIF-8, HFH@ZIF-8 and HFH@ZIF-8 + O_2_, respectively. (f) SDS-PAGE protein analysis of Hb, HFH@ZIF-8 and FH@ZIF-8. UV–vis absorption spectra (g) and fluorescence emission spectra (h) of HMME, HMME@ZIF-8, FH@ZIF-8, HFH@ZIF-8 and HFH@ZIF-8 + O_2_. (i) The DPBF absorption spectra in the presence of FH@ZIF-8, HFH@ZIF-8 and HFH@ZIF-8 + O_2_ under US irradiation.

HFH@ZIF-8 nanoparticles were firstly prepared by decorating HMME-loaded ZIF-8 nanoparticles (HMME@ZIF-8) with F127. And then F127-modified HMME@ZIF-8 (FH@ZIF-8) was mixed with hemoglobin and modified by electrostatic adsorption to produce HFH@ZIF-8 (Fig. 2(a)) while oxygen-carrying HFH@ZIF-8 were denoted as the status of HFH@ZIF-8 + O_2_. The modification of F127 was able to improve the hydrophilicity of HMME@ZIF-8, so that the nanoparticles can be fully dissolved in the water and be stored in a stable status [39]. The encapsulation efficiency of HFH@ZIF-8 is around 68.86 ± 2.96% (Fig. S1). Meanwhile, the presence of hemoglobin can provide the source of supplied oxygen during SDT [40]. As displayed by TEM image (Fig. 2(b) and 2(c)), FH@ZIF-8 demonstrated the hexagonal structure and uniform sizes while HFH@ZIF-8 exhibited slightly increased particle sizes and rougher surface. The prepared ZIF-8, HMME@ZIF-8, FH@ZIF-8, HFH@ZIF-8 and oxygen-carrying HFH@ZIF-8 were measured with dynamic light scattering (DLS), showing the average diameter was 24.4 nm, 50.7 nm, 58.8 nm, 91.3 nm and 106 nm, respectively (Fig. 2(d), S2). Likewise, the zeta potential of nanoparticles also changed significantly after the modification of hemoglobin and loading of oxygen (Fig. 2(e)). In particular, to quantify the protein decoration of nanoparticles, SDS-PAGE was carried out, in which the analysis results demonstrated that the protein band of HFH@ZIF-8 showed a very similar pattern with that of pure hemoglobin (Fig. 2(f)). In addition, the UV–vis absorption spectrum of HFH@ZIF-8, nanoparticles showed a characteristic absorption peak of HMME at the wavelength of 410 nm (Fig. 2(g)), indicating the successful loading of HMME. Further, as displayed in the fluorescence spectrum (Fig. 2(h)), HFH@ZIF-8 illustrated an obvious fluorescence emission around 820 nm, representing a significant red shift of that of the pure HMME. This might be because the loading HMME into ZIF-8 nanoparticles avoided the self-aggregation effect of HMME [41]. More importantly, a redox capacity test was used to inspect whether hemoglobin in the nanoparticles still has the function of carrying and releasing oxygen. As displayed by the UV absorption spectroscopy, HFH@ZIF-8 + O2 showed a characteristic absorption peak of oxyhemoglobin and HMME at the wavelength of 410 nm. After reduction by sodium sulfide (Na2S), the absorption peak of the nanoparticle solution was red-shifted to around 420 nm due to the transition of oxyhemoglobin into deoxyhemoglobin. Subsequently, oxygen was added into the solution again to ensure that hemoglobin in nanoparticles was oxidized, illustrating that the absorption spectra of HFH@ZIF-8 + O2 showed no significant change (Fig. S3). In addition, the particle size of HFH@ZIF-8 and HFH@ZIF-8 + O_2_ was respectively stable in PBS within 7 days (Fig. S4). In particular, various categories of nanoparticles with different concentrations of HMME showed no effect on cell viability of normal cells, such as macrophages RAW264.7 cells (Fig. S5).

Besides, the ROS production such as singlet oxygen (^1^O_2_), is a key measurement to access the efficiency of SDT. For the present work, DPBF was used to determine the capabilities of HFH@ZIF-8 as a nanosonosensitizer to produce ^1^O_2_ since its absorbance peak can be declined in the presence of ROS [37]. As plotted in Fig. 2i, the samples treated with the combination of ultrasonic stimulation (0.5 MHz, 1.2 W/cm2, DC = 50%, 10 min) and HFH@ZIF-8 + O_2_ exhibited the lowest absorption peaks as compared to those from other groups of nanoparticles, indicating that HFH@ZIF-8-mediated SDT indeed can effectively generate ^1^O_2_. More importantly, the presence of oxygen further enhanced the efficiency for the generation of ^1^O_2_. The efficient ^1^O_2_ production suggested that HFH@ZIF-8 is a potential nanosonosensitizer against bacterial infections.

### 3.2 Antibacterial effect of HFH@ZIF-8 mediated by SDT

**Fig. 3:**
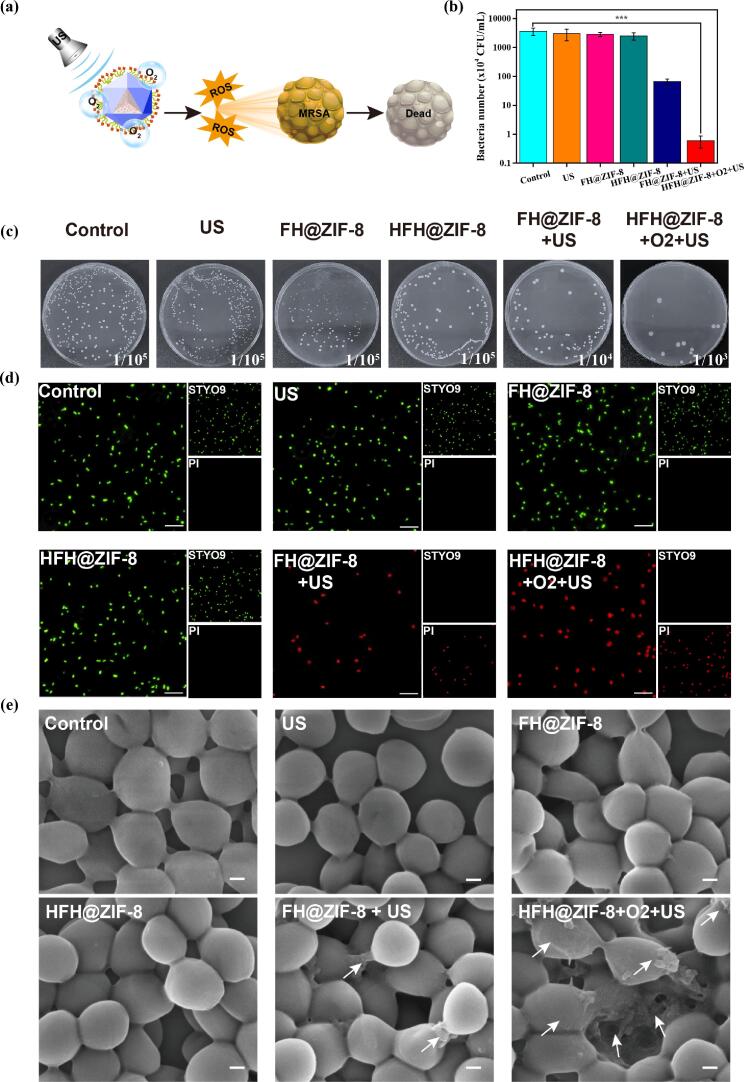
(a) Schematic of hemoglobin carrying oxygen to ensure US-irradiated HMME could enhance the production of reactive oxygen species to kill bacteria. (b) Viability of MRSA under different treatments. (c) Representative results of MRSA colonies on agar plates after treatment with PBS, US, FH@ZIF-8, HFH@ZIF-8 and FH@ZIF-8 + US, and HFH@ZIF-8 + O_2_ + US, repectively. (d) STYO9/PI staining of MRSA after different treatments. Viable MRSA (green) were stained with STYO9, and dead MRSA (red) were stained with PI. Scale bar:15 μm. (e) SEM images of MRSA treated with PBS, US, FH@ZIF-8, HFH@ZIF-8 FH@ZIF-8 + US, and HFH@ZIF-8 + O_2_ + US, respectively. Scale bar: 200 nm. White arrows indicate the wrinkled surface of MRSA after SDT.

MRSA as the representative bacteria was used to inspect the efficacy of antibacterial SDT via the colony counting method (Fig. 3(a)). In the absence of ultrasonic irradiation (0.5 MHz, 1.2 W/cm^2^, DC = 50%, 10 min), the treatments groups by using various nanoparticles including HF@ZIF-8 and HFH@ZIF-8 show no bactericidal activity (Fig. 3(b)). By contrast, the HFH@ZIF-8 treatment group with ultrasonic irradiation showed significant inhibition of the vitality of MRSA, demonstrating few numbers of colonies left on the agar plate (Fig. 3(c)). The fluorescence microscope imaging results with SYTO 9/PI staining assay also illustrated that US activation is essential for killing MRSA with HFH@ZIF-8 (Fig. 3(d)). Obviously, almost all live MRSA showed green fluorescence for the control group, US only treatment group, and different nanoparticles treatment groups without US. However, only the SDT treatment group mediated by HFH@ZIF-8, showed red fluorescence for dead MRSA as the bacterial cell membrane was destroyed by ROS and the internalization of PI was enhanced.

More specifically, MRSA as Gram-positive bacteria with a thick and porous cell wall can easily alleviate the treatment effect from the foreign drugs on the bacteria. To further explore the physiological mechanism of bacteria disruption due to HFH@ZIF-8 mediated by SDT, scanning electron microscopy (SEM) was used to detect the changes of MRSA morphology during SDT. It was discovered from Fig. 3(e) that the untreated bacteria had independent spherical cells structure with a smooth surface. This is not the case for the treatment group with HFH@ZIF-8-mediated by SDT, in which significant changes in the shapes and sizes of the bacteria were identified including the wrinkled and lysed bacterial cell wall as well as tiny and irregular bacterial fragments [42]. These observations demonstrated that the bacterial cell wall and cell membrane might be damaged due to the presence of ROS, which can inhibit the growth of MRSA.

### 3.3 *In vivo* biodistribution of HFH@ZIF-8

**Fig. 4:**
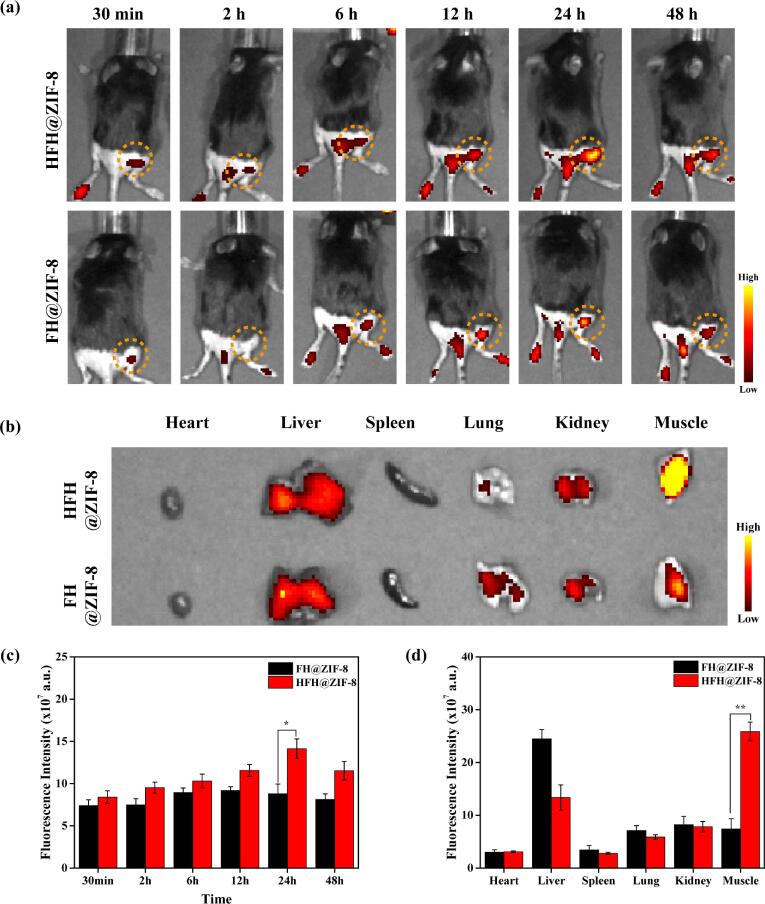
(a) *In vivo* fluorescence imaging of the mice treated with HFH@ZIF-8 and FH@ZIF-8 (5 mg/kg), respectively, at different time points (30 min, 2 h, 6 h, 12 h, 24 h and 48 h). (b) *Ex vivo* fluorescent imaging of major organs and infected muscles extracted from mice at 24 h post-injection. Semiquantitative biodistribution of FH@ZIF-8 and HFH@ZIF-8 quantified for the whole body at different time points *in vivo* (c) or for various organs at 24 h post-injection (d).

To detect the *in vivo* distribution and inflammation-targeting property of HFH@ZIF-8, FH@ZIF-8 and HFH@ZIF-8 nanoparticles were injected intravenously into the MRSA myositis-bearing mice and imaged by an *in vivo* spectrum imaging system (IVIS) at different time points. The fluorescence was due to the HMME that were loaded into the nanoparticles. As shown in Fig. 4(a), HFH@ZIF-8 exhibited the efficient inflammation-targeting ability in MRSA myositis, emitting strong fluorescence at 2 h post-injection and reaching the peak at 24 h. Enhanced strong fluorescence can be detected at the myositis site along with time from the mice administrated with HFH@ZIF-8 although this is not the case for the control mice injected with the FH@ZIF-8. In addition, MRSA injected into the legs also caused footpad infections, leading to the accumulation of nanoparticles and increased fluorescent signal on footpads. To image and quantify the fluorescence signals in various organs, mice were euthanized 24 h post-injection and the harvested organs and MRSA-infected leg were imaged. It was discovered that FH@ZIF-8 were mainly detected in the liver and spleen. By contrast, the accumulation of HFH@ZIF-8 in the liver was much less as compared to that from the control group (Fig. 4(b)). However, significantly higher accumulation in the MRSA-infected leg was identified for the HFH@ZIF-8 case. The quantitative analysis revealed a 1.6-fold increase of relative fluorescence intensity in the MRSA-infected leg associated with HFH@ZIF-8 treatment group in comparison with that of FH@ZIF-8, being consistent with findings from *in vivo* fluorescence imaging (Fig. 4(c-d)).

### 3.4 Treatment of MRSA-induced myositis *in vivo*

**Fig. 5:**
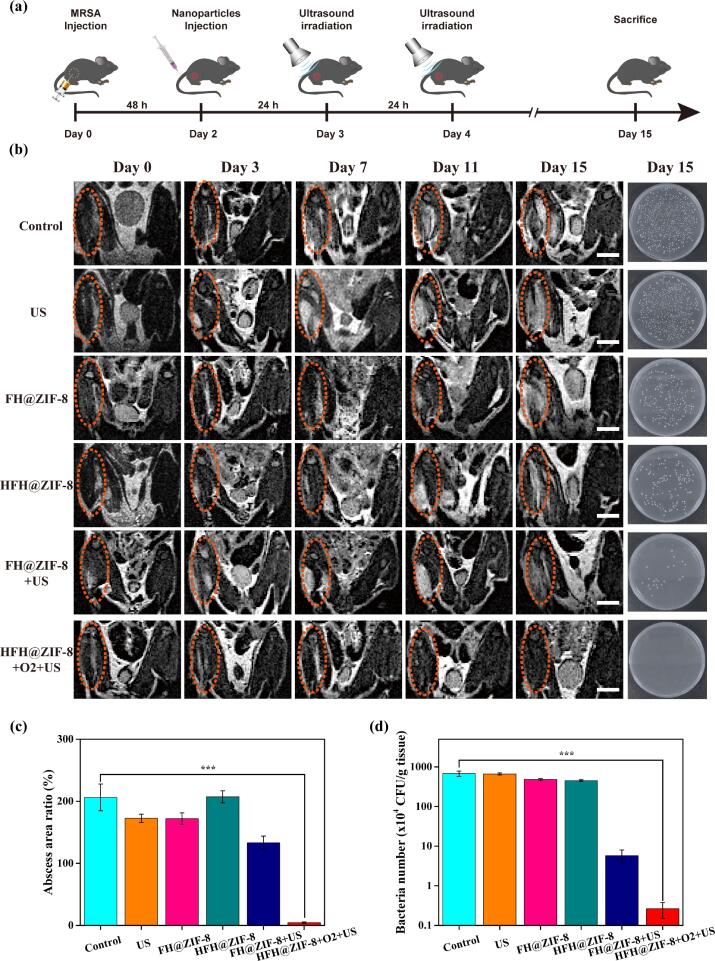
(a) Schematic of the bacterial infection and US treatment areas in mice with MRSA myositis. (b) Representative MRI images for monitoring MRSA-infected mice for different treatment groups in 14 days. Bar = 0.5 cm. On the middle right (Fig. 5(b)) were the counting forming unit of MRSA harvested from different treatment groups. (c-d) Quantitative features of infected areas quantified from MRI results and the survival rate of MRSA of infection muscles with various treatment strategies.

To access the *in vivo* anti-bacteria effect of HFH@ZIF-8, a MRSA-induced myositis-bearing mice model was developed for carrying out SDT according to the therapeutic protocol in Fig. 5(a). The mice were randomly separated into six treatment groups: (1) saline (control), (2) ultrasound alone (US), (3) FH@ZIF-8 alone (FH@ZIF-8), (4) HFH@ZIF-8 alone (HFH@ZIF-8), and (5) FH@ZIF-8 + US group or HFH@ZIF-8 + O_2_ + US group, respectively. US irradiation (0.5 MHz, 1.2 W/cm^2^, 50% duty cycle, 10 min) was performed at 24 h and 48 h after intravenous tail-vein injection of different nanoparticles to trigger the SDT. Then, small animals MRI was conducted to visualize and monitor the progression of myositis during SDT.

As shown in Fig. 5 (b), the captured MRI features for bacterial infection muscular edema area (dot curve marked areas) in mice legs were very similar for each treatment group on day 0. However, when the duration of SDT was up to 15 days, HFH@ZIF-8 + US treatment group exhibited the most significant inhibitory effect against MRSA infection and the smallest edema area, whereas other treatment groups with saline, FH@ZIF-8 alone or HFH@ZIF-8 alone were not able to suppress inflammation development, demonstrating a significant tissue deterioration from diffuse muscle edema to focal liquefaction abscess cavity. Basically, the FH@ZIF-8 + US treatment slightly alleviated the MRSA infection through SDT (Fig. 5(c)). In addition, to detect the antibacterial efficacy of the infection treatment, the infected muscle tissue was collected, homogenized, diluted (10000-fold), and plated on Luria-Bertani (LB) plates to evaluate the MRSA count in 15 days after SDT. Interestingly, it was discovered that the average bacterial colony number of mice treated with HFH@ZIF-8 + O_2_ + US was<5 × 10^3^ CFU/g tissue, whereas that of control groups reached up to 5 × 10^6^ CFU/g tissue, indicating that the infection disease recovery due to MRSA was very effective for the HFH@ZIF-8 + O_2_ + US group (Fig. 5(d)).

**Fig. 6:**
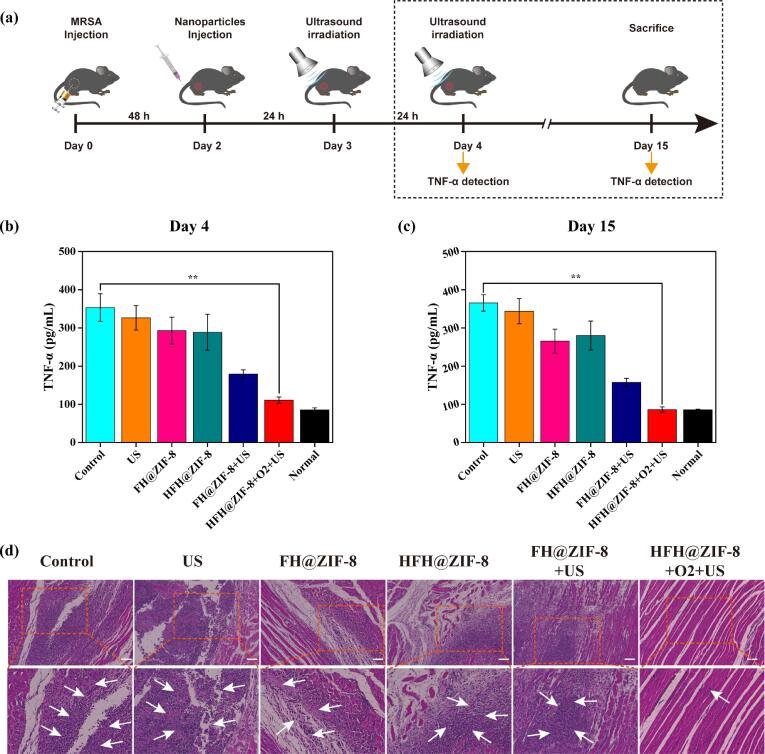
(a) Schematic diagram of the cytokines detection in mice with MRSA myositis. The level of TNF-α in serum from mice at Day 4 (b) and Day 15 post injection (c). (d) Muscular tissue histology after H&E staining. Scale bar: 100 μm. The arrows in histological images indicated the inflammatory cell infiltration, fibrous tissue hyperplasia and abscess cavity formation.

To understand the impact of HFH@ZIF-8-mediated SDT on the inflammatory response *in vivo*, enzyme-linked immunosorbent assay (ELISA) was used to quantitatively analyze the levels of pro-inflammatory cytokines TNF-α in mice serum (Fig. 6(a)). The serum TNF-α levels of mice in the control group were significantly higher than those in the normal group on day 4, whereas the HFH@ZIF-8-mediated SDT treatment group only demonstrated a slight increase in cytokine levels. However, the TNF-α levels of the US, FH@ZIF-8, and HFH@ZIF-8 treatment groups were similar to those of the control group. On the day 15, the TNF-α levels of each group were slightly lower than before due to the influence of intrinsic immune response [43]. Except the HFH@ZIF-8 + US treatment group, the cytokine levels of other groups were still significantly higher than the normal group (Fig. 6(b-c)). Further, the lesion and healed muscle tissue were evaluated by using histopathological examination. H&E-staining slices findings in Fig. 6(d) demonstrated that only mice treated with HFH@ZIF-8 + US showed normal muscle tissue texture, while the muscle tissues of the other groups exhibited obvious inflammatory cell infiltration, fibrous tissue hyperplasia and abscess cavity formation.

### 3.5 Biological safety of HFH@ZIF-8

**Fig. 7:**
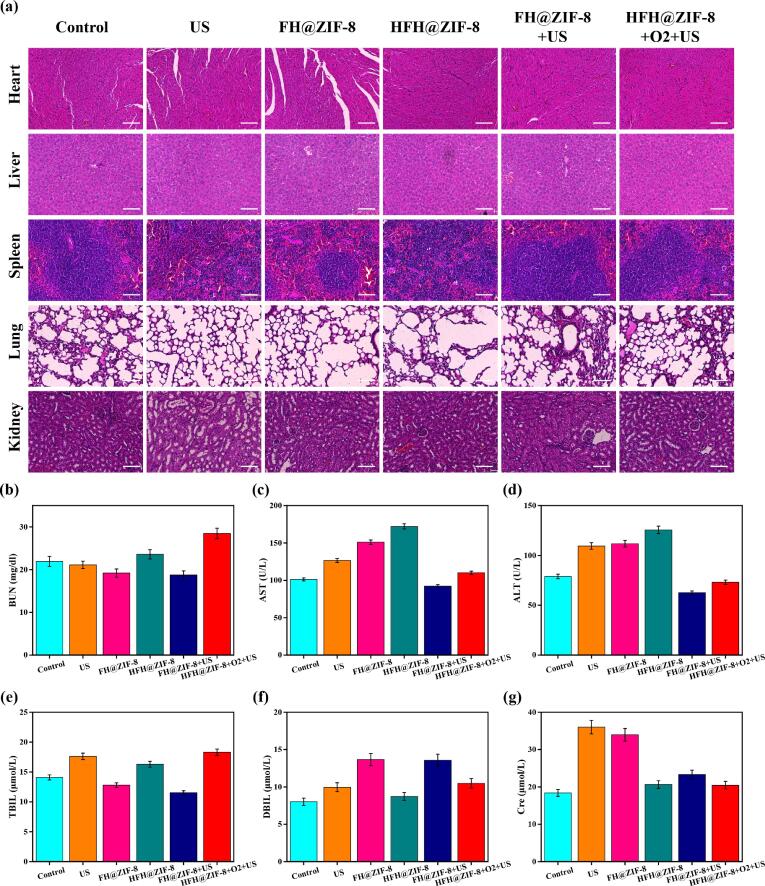
(a) H&E staining images of major organs extracted from MRSA infection mice after different treatments. (b-g) Serum biochemical indicators of mice with different treatments.

To further inspect the biosafety of various therapeutic strategies, the histopathologic features of major organs extracted from mice body were analyzed (Fig. 7(a)). It was discovered that no obvious cell damages were detected in the H&E staining organs slices of mice from different treatment groups. In addition, the blood biochemistry and blood routine data were acquired, demonstrating that all the indexes of HFH@ZIF-8 + O_2_ + US treatment group exhibited no significant difference from those of the other treatment groups (Fig. 7(b-g), Table S1). Further, we also examined the hemolytic activity of HFH@ZIF-8 nanosonosensitizer on red blood cells (Fig. S6). It was discovered that when the concentration was up 300 μg/ml, no obvious hemolysis was identified, indicating that HFH@ZIF-8 nanosonosensitizer as a potential nanodrug has excellent blood compatibility and superior biosafety.

## 4 Conclusion

In this study, a new nanosonosensitizer (HFH@ZIF-8) for SDT was constructed for killing bacteria and treatment of *in vivo* infection diseases. In particular, the HFH@ZIF-8 exhibited the inflammation-targeting capabilities and reliable MRSA-suppressive effects upon their exposure to US. More importantly, this developed treatment strategy allows for enhancing the ROS generating efficiency for *in vivo* SDT by the delivery and supply of oxygen by hemoglobin. Therefore, the novel targeted nanotheranostic platform that combines HFH@ZIF-8 with US holds great promise for bacterial infection treatment in in deep tissue.

## Declaration of Competing Interest

The authors declare that they have no known competing financial interests or personal relationships that could have appeared to influence the work reported in this paper.
