# Supplementary material for: Oxygen-carrying biomimetic nanoplatform for sonodynamic killing of bacteria and treatment of infection diseases
Source: Ultrason Sonochem. 2022 Mar 2;84:105972. doi: 10.1016/j.ultsonch.2022.105972 (PMC8897654; doi:10.1016/j.ultsonch.2022.105972)
Supplement: Supplementary data 1 [file mmc1.docx]

**Supporting Information**

**Oxygen-Carrying Biomimetic Nanoplatform for Sonodynamic Killing of Bacteria and Treatment of Infection Diseases**

Xiaorui Geng^1,#^, Yuhao Chen^1,#^, Zhiyi Chen^2,3,#^, Xianyuan Wei^1^, Yunlu Dai^1^, Zhen Yuan^1,4^

^1^ Cancer Center, Faculty of Health Sciences, University of Macau, Taipa, Macau SAR, China

^2^ The First Affiliated Hospital, Medical Imaging Centre, Hengyang Medical School, University of South China, Hengyang, Hunan, China

^3^ Institute of Medical Imaging, Hengyang Medical School, University of South China, Hengyang, China

^4^ Centre for Cognitive and Brain Sciences, University of Macau, Taipa, Macau SAR , China

* Corresponding author: E-mail: zhenyuan@um.edu.mo; # Equal Contribution

**Supporting figures**


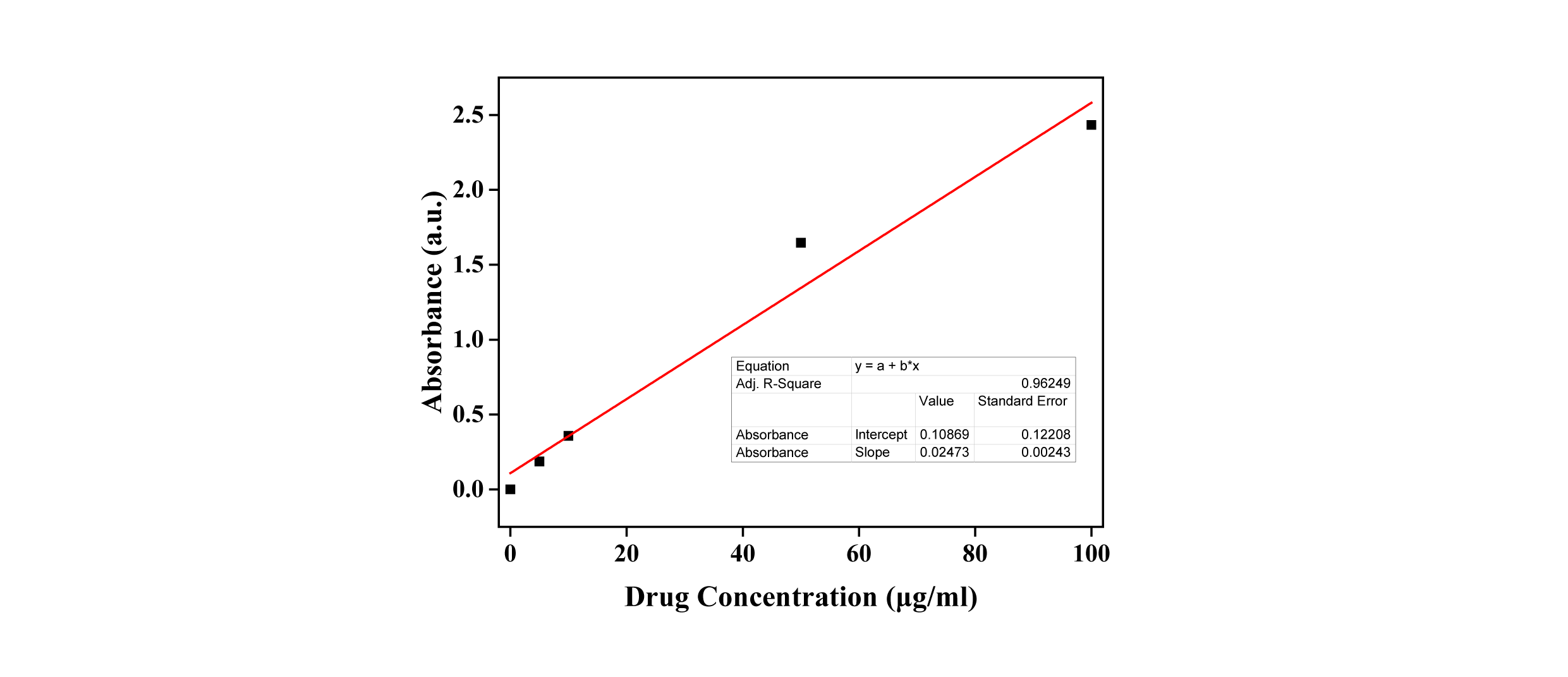


**Fig. S1** Linear dependence of HMME absorbance intensity on its concentration changes from 0-100 μg/mL.


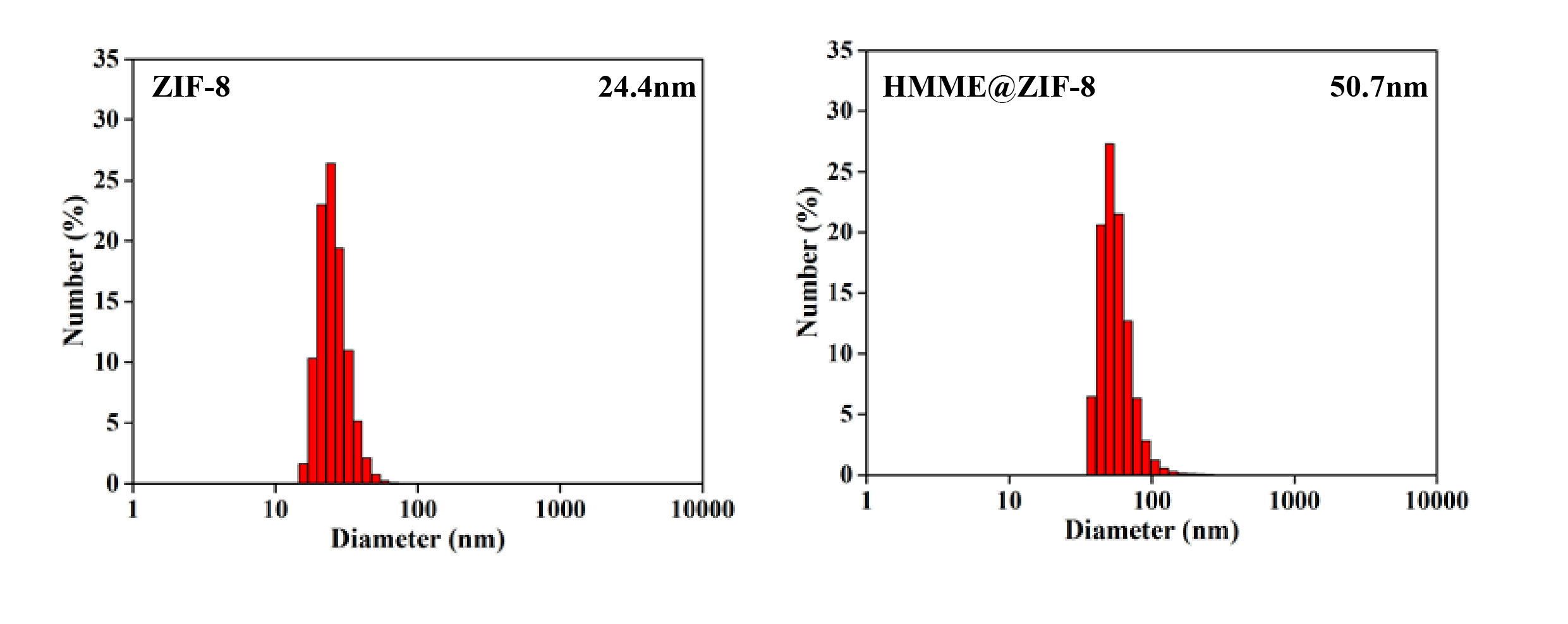


**Fig. S2** The particle size of ZIF-8 and HMME@ZIF-8, respectively.


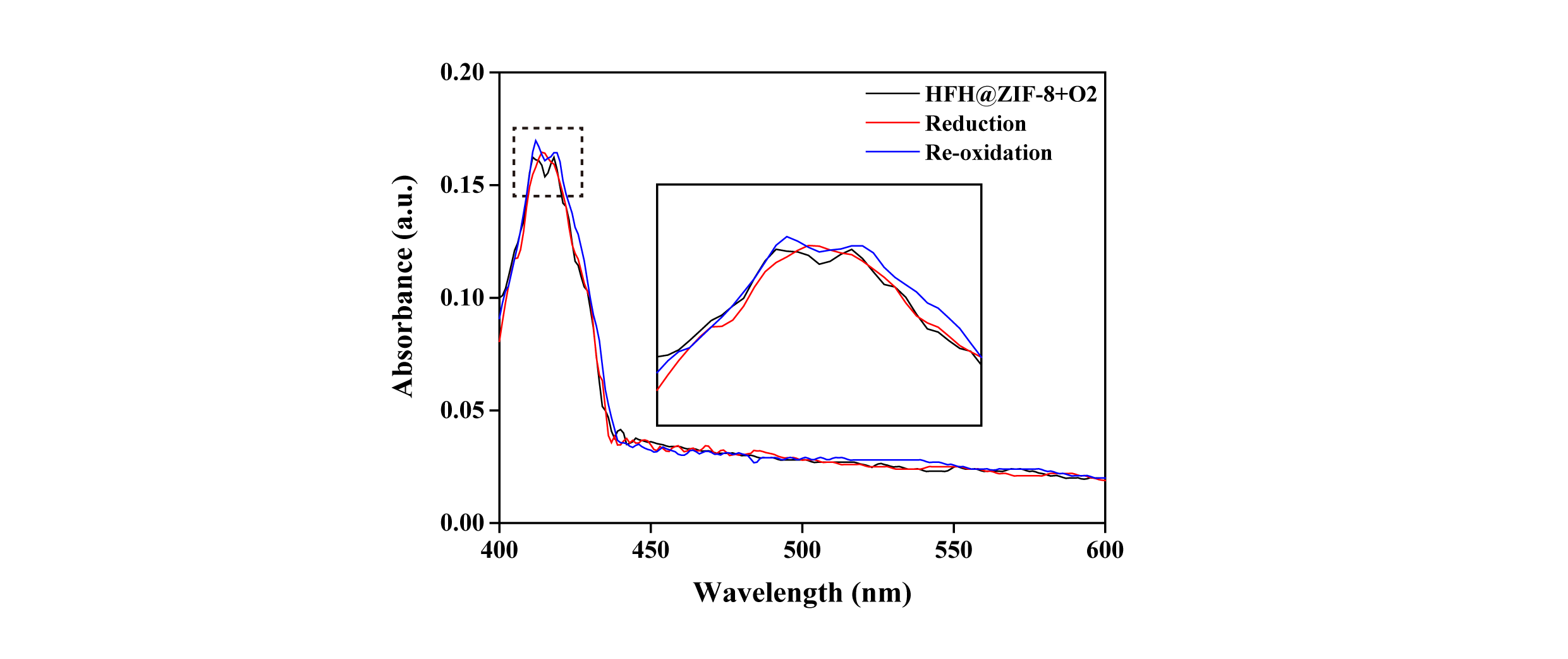


**Fig. S3** Redox activity of HFH@ZIF-8+O_2_ illustrated by the shift of absorption spectrum.


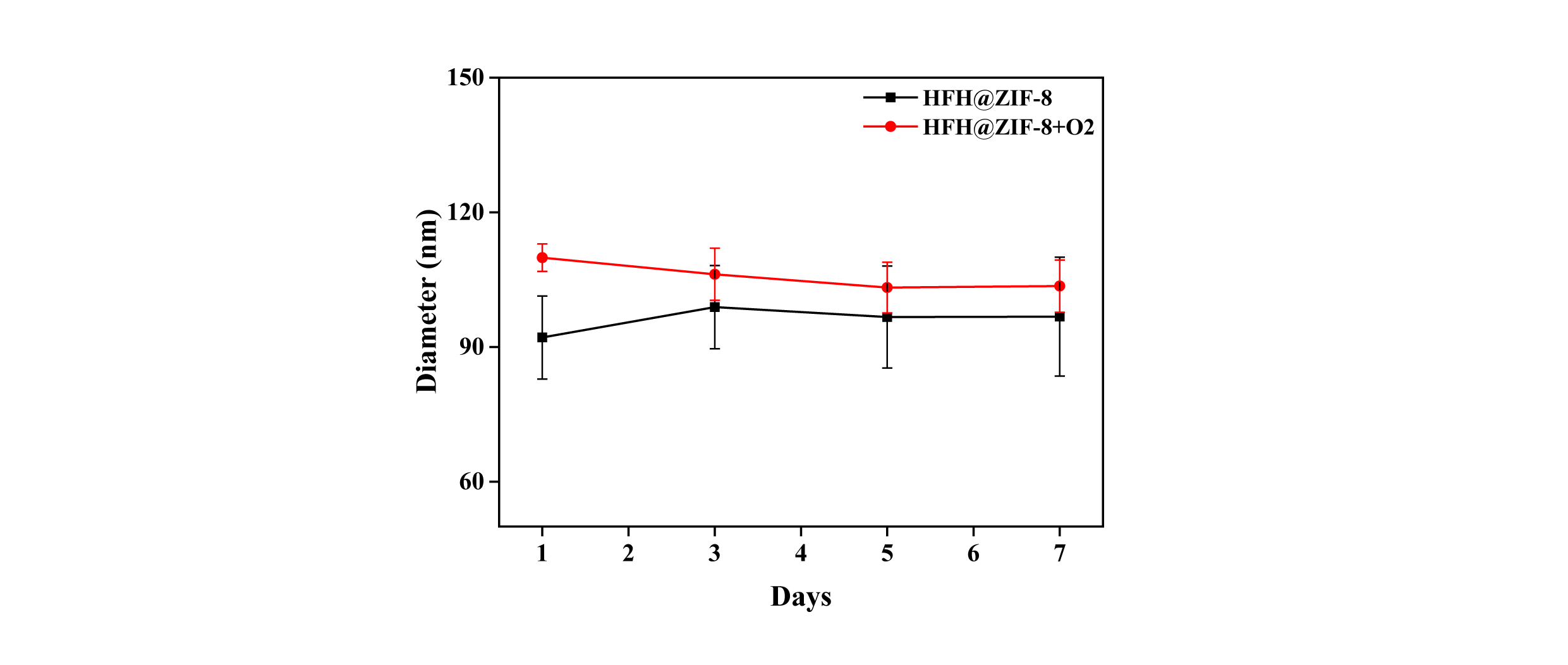


**Fig. S4** The stability of HFH@ZIF-8 and HFH@ZIF-8+O_2_ in PBS, respectively.


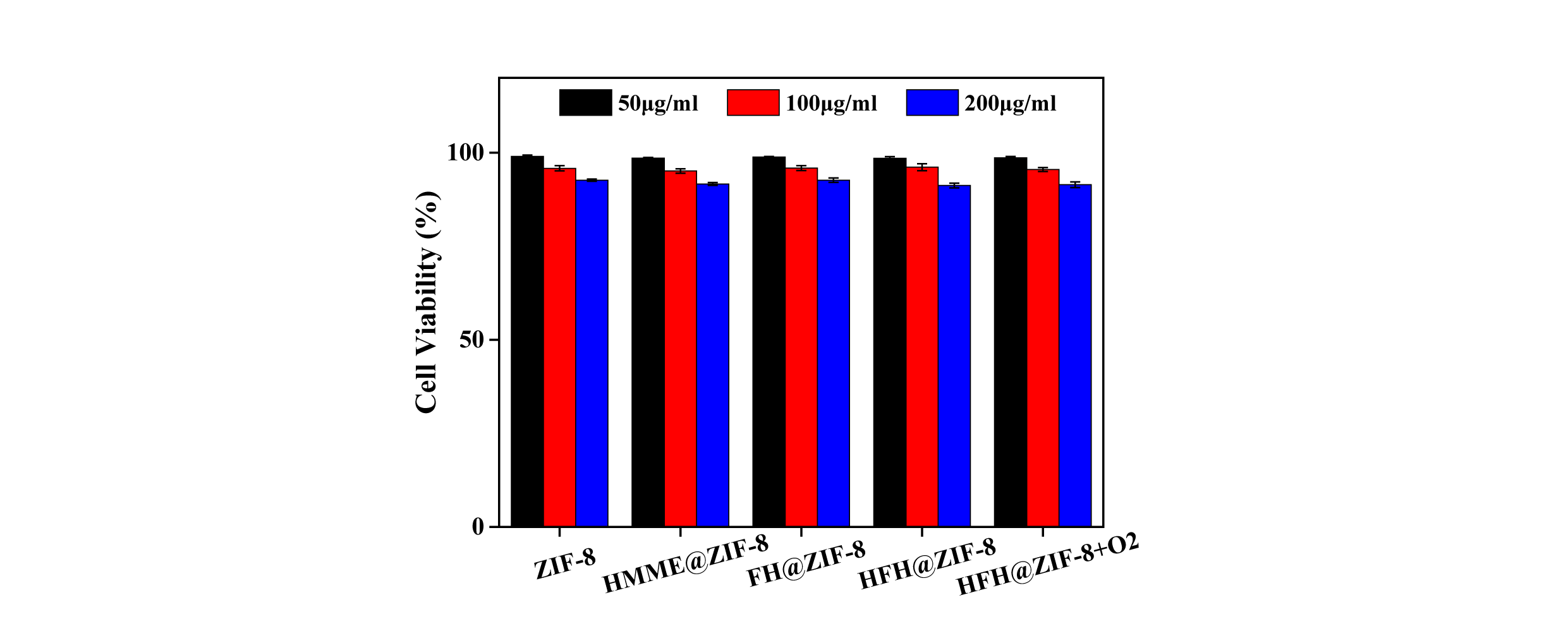


**Fig.S5** Cytotoxicity of various categories of nanoparticles with different concentrations of HMME.


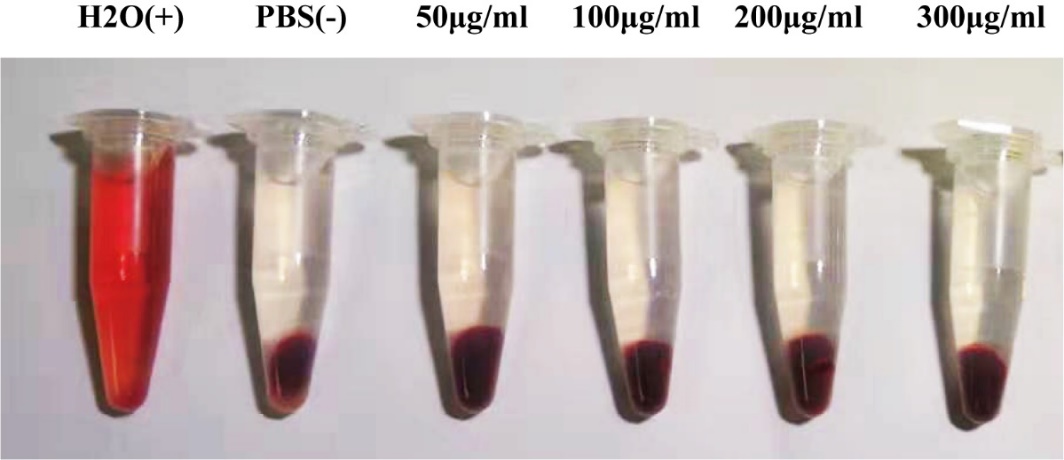


**Fig. S6** The hemolysis of red blood cells after treated with different concentrations of HFH@ZIF-8 for 3h.

**Supporting table**

Table S1. Blood routine of mice from the in vivo treatment experiments.

|  | Control | US | FH@ZIF-8 | HFH@ZIF-8 | FH@ZIF-8  + US | HFH@ZIF-8+O2+US |
| --- | --- | --- | --- | --- | --- | --- |
| WBC (10^9^/L) | 6.3±0.4 | 6.1±0.5 | 6.6±0.4 | 6.3±0.3 | 5.2±0.6 | 6.0±0.4 |
| Lymph#(109/L) | 3.3±0.2 | 4.9±0.4 | 5.1±0.6 | 5.6±0.5 | 4.2±0.3 | 5.5±0.6 |
| Mon# (109/L) | 0.3±0.05 | 0.2±0.06 | 0.2±0.04 | 0.2±0.03 | 0.2±0.05 | 0.3±0.06 |
| Gran# (109/L) | 1.7±0.15 | 1.2±0.23 | 1.3±0.31 | 1.2±0.16 | 1.6±0.24 | 1.2±0.18 |
| Lymph% (%) | 56.9±5.9 | 78.1±6.5 | 77.1±6.7 | 80.7±4.2 | 75.3±5.4 | 68.6±3.6 |
| Mon% (%) | 4.2±0.5 | 2.5±0.4 | 2.7±0.7 | 2.3±0.2 | 2.8±0.6 | 3.5±0.3 |
| Gran%(%) | 32.9±1.6 | 19.4±5.7 | 20.2±4.3 | 17.0±2.9 | 21.9±3.7 | 27.9±4.8 |
| RBC (1012/L) | 8.55±1.62 | 9.16±2.76 | 8.17±2.29 | 10.20±3.13 | 8.62±3.55 | 7.37±2.17 |
| HGB (g/L) | 132±8.6 | 140±5.2 | 129±10.6 | 136±7.5 | 130±4.9 | 142±8.1 |
| HCT (%) | 42.9±5.9 | 42.0±6.6 | 35.8±8.1 | 37.2±7.3 | 41.0±4.5 | 43.8±4.9 |
| MCV (fl) | 50.0±5.6 | 49.2±8.1 | 49.1±6.3 | 48.3±4.2 | 49.2±5.7 | 50.2±5.3 |
| MCH (pg) | 16.8±2.1 | 16.0±1.8 | 16.6±2.4 | 18.3±3.3 | 16.1±4.0 | 16.6±3.1 |
| MCHC (g/L) | 307±34.1 | 317±29.4 | 304±19.5 | 309±24.6 | 306±21.8 | 303±24.3 |
| RDW (%) | 15.3±0.86 | 16±0.45 | 16.7±0.71 | 15.6±0.62 | 16.3±0.71 | 16.0±1.04 |
| PLT (109/L) | 1014±294.6 | 1490±237.4 | 1370±197.2 | 1057±186.1 | 1330±246.8 | 1498±108.4 |
| MPV (fl) | 5.8±0.4 | 5.9±0.1 | 5.9±0.2 | 5.1±0.3 | 5.9±0.1 | 6.4±0.2 |
| PDW (%) | 16.1±2.2 | 16.1±3.8 | 15.8±1.8 | 16.3±1.9 | 16.0±2.5 | 16.2±2.4 |
